# Optimization of diagnostic performance for differentiation of recurrence from radiation necrosis in patients with metastatic brain tumors using tumor volume-corrected ^11^C-methionine uptake

**DOI:** 10.1186/s13550-017-0293-0

**Published:** 2017-05-23

**Authors:** Tae-Young Jung, In-Young Kim, Sa-Hoe Lim, Ki Seong Park, Dong-Yeon Kim, Shin Jung, Kyung-Sub Moon, Woo-Youl Jang, Sae-Ryung Kang, Sang-Geon Cho, Jung-Joon Min, Hee-Seung Bom, Seong Young Kwon

**Affiliations:** 1 Department of Neurosurgery, Chonnam National University Hwasun Hospital, 322 Seoyang-ro, Hwasun-eup, Hwasun-gun, Jeonnam 58128 Republic of Korea; 2 Department of Nuclear Medicine, Chonnam National University Hwasun Hospital, 322 Seoyang-ro, Hwasun-eup, Hwasun-gun, Jeonnam 58128 Republic of Korea

**Keywords:** ^11^C-methionine PET/CT, Metabolic tumor volume, Metastatic brain tumor, Recurrence, Radiation necrosis

## Abstract

**Background:**

Tumor to normal tissue ratio (T/N ratio) on ^11^C-methionine (^11^C-MET) positron emission tomography/computed tomography (PET/CT) is affected by variable factors. We investigated whether T/N ratio cutoff values corrected according to metabolic tumor volume (MTV) could improve the diagnostic performance of ^11^C-MET PET/CT for diagnosis of recurrence in patients with metastatic brain tumor.

Forty-eight patients with metastatic brain tumors underwent ^11^C-MET PET/CT for differential diagnosis between recurrence and radiation necrosis after gamma knife radiosurgery (GKR). Both T/N ratio and MTV were estimated in each lesion on ^11^C-MET PET/CT. The lesions were classified into three groups based on MTV criteria (≤ 0.5 cm^3^; > 0.5, ≤ 4.0 cm^3^; and > 4.0 cm^3^). The optimal cutoff values of the T/N ratio from receiver operating characteristic (ROC) curve were determined in each group (MTV-corrected) as well as total lesions (non-corrected). Finally, diagnostic performance of ^11^C-MET PET/CT was compared with the MTV-corrected cutoff values.

**Results:**

Among 77 lesions, 51 were diagnosed with recurrence. The mean T/N ratio was 2.25 (± 1.12) for recurrent lesions and 1.44 (± 0.22) for radiation necrosis (*P* < 0.001). T/N ratio of 1.61 (non-corrected) provided the best sensitivity, specificity, and diagnostic accuracy (70.6, 80.8, and 74.0%, respectively). Using the MTV criteria, optimal cutoff values of the T/N ratios in each group were 1.23 (MTV ≤ 0.5 cm^3^), 1.54 (0.5 cm^3^ < MTV ≤ 4.0 cm^3^), and 1.85 (MTV > 4.0 cm^3^). In small-sized lesions (MTV ≤ 0.5 cm^3^), MTV-corrected cutoff values (1.23) could maintain favorable diagnostic performance with sensitivity, specificity, and diagnostic accuracy (70.0, 80.0, and 73.3%, respectively), compared to non-corrected cutoff values.

**Conclusions:**

MTV-corrected cutoff values of T/N ratio could maintain the diagnostic performance of ^11^C-MET PET/CT in small sized, metastatic brain tumors. We expect our results to contribute to reproducible and standardized interpretation of ^11^C-MET PET/CT.

## Background

^11^C-methionine (^11^C-MET) positron emission tomography/computed tomography (PET/CT) has been used to diagnose primary and metastatic brain tumors [1–3]. Tumor to normal tissue ratio (T/N ratio) is a useful metabolic parameter to differentiate tumor recurrence from radiation necrosis after gamma knife radiosurgery (GKR) [2], which is an important treatment modality for metastatic brain tumors [4].

The T/N ratio is a relative index that can be affected by variable factors. Previous studies have shown that the cutoff value of T/N ratio for differentiation of tumor recurrence from radiation necrosis depends on the histologic types, tumor sizes, and other parameters [2, 5, 6]. T/N ratio could especially be affected by tumor size because ^11^C-MET uptake in small-sized tumors is underestimated through a partial volume effect (PVE) [5, 7]. Although a different cutoff value of the T/N ratio should be applied for diagnosis according to tumor size, there have been no studies regarding reference parameters for measuring tumor sizes to stratify the cutoff values of T/N ratio.

Metabolic tumor volume (MTV) usually represents the volume of metabolically active tumor in a reproducible manner [8, 9], which could be a potential reference parameter to measure tumor size. In this study, we investigated whether cutoff values of the T/N ratio can be stratified according to MTV and the resulting improvement in the diagnostic performance of ^11^C-MET PET/CT for diagnosis of tumor recurrence in patients with metastatic brain tumors.

## Methods

### Patient evaluation

Initially, we included 68 patients with metastatic brain tumors referred for evaluation of recurrence after GKR using ^11^C-MET PET/CT from November 2008 to June 2015. Out of these, 20 patients with insufficient follow-up were excluded. Finally, 48 patients who underwent ^11^C-MET PET/CT due to clinical symptoms or abnormal magnetic resonance imaging (MRI) findings were enrolled in the study. A retrospective review of medical records was performed for these patients. The mean age was 62.0 years (range 45–75 years), and included both men (*n* = 26) and women (*n* = 22). The primary tumor site was the lungs in 38 patients, breasts in eight, esophagus in one, and uterine cervix in one. Pathological examination of the lung showed adenocarcinoma in 23 patients, small cell carcinoma in eight, squamous cell carcinoma in three, and others in four. In GKR, the median tumor volume on MRI was 7.3 cm^3^ (range 1.2–21.8 cm^3^), and the median prescription dose was 18.0 Gy (range 14–22 Gy). The median duration from GKR to ^11^C-MET PET/CT examination was 11.3 months (range 2.1–49.1 months). Repeated GKR was performed in patients with suspected recurrence based on clinical symptoms, MRI, and ^11^C-MET PET/CT images.

Recurrence was diagnosed in cases of pathological confirmation or tumor control after repeated GKR over 4 months with radiological follow-up. Radiation necrosis was diagnosed in cases of pathological confirmation or stable or reduced volume without treatment for 4 months with radiological follow-up. The treatment did not include chemotherapy to control the primary tumor. This retrospective study was approved by our institutional review board.

### ^11^C-MET PET/CT

PET/CT was performed using a Discovery ST PET/CT system (GE Medical Systems, Milwaukee, WI, USA), with a spatial resolution of 5.0 mm (full width at half maximum) and slice thickness of 3.27 mm. Patients were positioned in the scanner such that slices parallel to the orbitomeatal line could be obtained. Patients were intravenously injected with ^11^C-MET at 7 MBq/kg during a fasting period. For attenuation correction, we acquired a non-contrast-enhanced, low-dose CT scan and began a 10-min emission scan, 20 min after injecting ^11^C-MET. We reconstructed transaxial images of 256 × 256 × 98 anisotropic voxels (voxel size was 1.17 mm × 1.17 mm × 3.27 mm) with ordered subset expectation maximization (OSEM: iteration, 5; subset: 32) and used CT images for attenuation correction of the PET images.

### Image analyses

For the evaluation of ^11^C-MET uptake in the tumor, volumetric region of interest (VOI) was placed covering the entire tumor, and the highest value of VOI was selected (maximum standardized uptake value, SUVmax). For reference, fixed spherical VOI (844 mm^3^) of about 5.86 mm radius was placed on the gray matter of the contralateral frontal lobe; if this was not possible because of the tumor location, it was placed on intact brain regions in the axial plane.

The T/N ratio was defined as SUVmax of the lesion divided by that of the reference VOI. MTV (cm^3^) was measured by applying a threshold of 1.3 times the mean ^11^C-MET uptake of contralateral normal gray matter [10]. Metabolic parameter measurement was performed on an Advantage Workstation (GE Healthcare) using the PET-VCAR (Volume Computer-Assisted Reading) software (version 1.0).

### Statistical analysis

Volume criteria for tumor grouping were induced by tumor diameter (1 cm diameter, MTV 0.5 cm^3^; 2 cm diameter, MTV 4.0 cm^3^) considering PVE [5]. Then, tumor lesions on ^11^C-MET PET/CT were classified into three groups based on MTV criteria (≤ 0.5 cm^3^; > 0.5, ≤ 4.0 cm^3^; and > 4.0 cm^3^). The optimal cutoff value of T/N ratio was determined from receiver operating characteristic (ROC) curve in each group. The diagnostic performance for all lesions was evaluated by combining diagnostic accuracy of each group after applying different cutoff values of T/N ratio (MTV-corrected cutoff values) and was compared with that of a single cutoff value regardless of MTV criteria (non-corrected cutoff value).

SPSS (version 21.0; IBM) was used for statistical analyses. Mann-Whitney *U* test was used for the comparison of T/N ratio between recurrence and radiation necrosis in each group categorized by MTV criteria. ROC curve analysis was used to determine the optimal cutoff values of the T/N ratio in each group based on MTV criteria as well as all lesions regardless of the criteria. McNemar’s test was used to observe the differences in diagnostic accuracies between the MTV-corrected and non-corrected cutoff values in each group. Descriptive statistics are presented as mean ± SD or as range. All statistical analyses were performed with a significance level of *P* < 0.05.

## Results

Among 77 lesions on ^11^C-MET PET/CT, 51 showed recurrence, which was diagnosed after pathologic confirmation in nine lesions and radiological tumor control after repeated GKR in 42 lesions. Twenty-six lesions were radiologically defined as radiation necrosis showing stable or reduced volume without treatment.

### Comparison of ^11^C-MET uptake between recurrence and radiation necrosis

In all lesions (n = 77), T/N ratio was significantly higher for recurrence than for radiation necrosis (2.25 ± 1.12 vs. 1.44 ± 0.22, *P* < 0.001). When we classified 77 lesions into three groups according to MTV criteria, 15 lesions were included in the MTV ≤ 0.5 cm^3^ group, 35 lesions in the 0.5 < MTV ≤ 4.0 cm^3^ group, and 27 lesions in the MTV > 4.0 cm^3^ group (Table 1). The T/N ratio from ^11^C-MET PET/CT was also higher for recurrence than for radiation necrosis in the three groups, although the mean values of T/N ratio in both recurrence and radiation necrosis tended to be lower as MTV decreased (Table 1).

**Table 1:** Comparison of ^11^C-methionine uptake according to metabolic tumor volume

MTV (cm^3^)	T/N ratio (mean ± SD (range))		*P* value
	Recurrence	Radiation necrosis	
≤ 0.5 (*n* = 15)	1.31 ± 0.10 (1.20–1.47)	1.19 ± 0.09 (1.08–1.33)	0.037
> 0.5, ≤ 4.0 (*n* = 35)	1.93 ± 0.61 (1.35–4.00)	1.47 ± 0.18 (1.21–1.88)	0.001
**>** 4.0 (*n* = 27)	2.92 ± 1.28 (1.62–5.89)	1.65 ± 0.16 (1.48–1.84)	0.008

*T/N ratio* tumor to normal tissue ratio, *MTV* metabolic tumor volume

### Different cutoff values for tumor to normal tissue ratio according to metabolic tumor volume

Using MTV criteria, optimal cutoff value of T/N ratio was defined in each group (Table 2). For lesions with an MTV ≤ 0.5 cm^3^, T/N ratio of 1.23 provided the best sensitivity and specificity (70.0 and 80.0%, respectively). In contrast, T/N ratio of 1.54 provided the best sensitivity and specificity (77.8 and 76.5%, respectively) for lesions with an MTV that ranged from 0.5 to 4.0 cm^3^. Finally, T/N ratio of 1.85 provided the best sensitivity and specificity (82.6 and 100%, respectively) for lesions with an MTV > 4.0 cm^3^. These results showed that different cutoff values of the T/N ratio based on tumor size (MTV-corrected cutoff values) should be applied for acceptable diagnostic accuracy on ^11^C-MET PET/CT.

**Table 2:** Different cutoff values for tumor to normal tissue ratio according to metabolic tumor volume

MTV (cm^3^)	AUC	Optimal cutoff value	Sensitivity (%)	Specificity (%)	PPV (%)	NPV (%)	Accuracy (%)
≤ 0.5 (*n* = 15)	0.840	1.23	70.0	80.0	87.5	57.1	73.3
> 0.5, ≤ 4.0 (*n* = 35)	0.817	1.54	77.8	76.5	77.8	76.5	77.1
**>** 4.0 (*n* = 27)	0.924	1.85	82.6	100	100	50	85.2

*MTV* metabolic tumor volume, *AUC* area under the ROC curve, *PPV* positive predictive value, *NPV* negative predictive value

### Comparison of diagnostic performance for recurrence by tumor volume-corrected cutoff values

Before comparison of diagnostic accuracy, optimal cutoff value of T/N ratio was defined in all lesions without MTV correction (non-corrected cutoff value), for which T/N ratio of 1.61 provided the best sensitivity, specificity, and diagnostic accuracy (70.6, 80.8, and 74.0%, respectively) (Table 3). However, in the group with MTV ≤ 0.5 cm^3^, there was no recurrent lesion with T/N ratio more than the non-corrected cutoff value (1.61). By applying MTV-corrected cutoff values of T/N ratio (1.23), diagnostic performance could be maintained without decreasing specificity in small-sized lesions (Table 2).

**Table 3:** Comparison of diagnostic accuracy for recurrence by tumor volume-corrected cutoff values

	Sensitivity (%)	Specificity (%)	PPV (%)	NPV (%)	Accuracy (%)
Non-corrected cutoff value (1.61)	70.6	80.8	83.7	61.8	74.0
MTV-corrected cutoff value	78.4	80.8	88.9	65.6	79.2

*PPV* positive predictive value, *NPV* negative predictive value, *MTV* metabolic tumor volume, *MTV-corrected optimal cutoff value* MTV ≤ 0.5 cm^3^—cutoff 1.23, 0.5 < MTV ≤ 4 cm^3^—cutoff 1.54, MTV > 4 cm^3^—cutoff 1.85

**Fig. 1:**
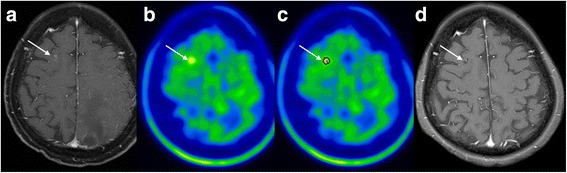
Imaging of a 75-year-old woman who underwent gamma knife radiosurgery (GKR) at a dose of 18 Gy for metastatic brain tumor that originated from lung adenocarcinoma. **a** T1-weighted MR image with gadolinium enhancement showing contrast-enhanced lesion in the right frontal lobe (*arrow*). **b** ^11^C-MET PET image showing mild focal tracer uptake corresponding to enhanced lesion on MR image (*arrow*). **c** Metabolic tumor volume (MTV) was 0.156 cm^3^ (*arrow*). T/N ratio was 1.29, which was lower than the non-corrected cutoff value (1.61) but more than the MTV-corrected cutoff value (1.23). **d** This lesion was determined as recurrence by definition, the size of which significantly decreased after repeated GKR over 4 months as observed in a follow-up MR image (*arrow*)

When we evaluated the diagnostic value of ^11^C-MET PET/CT for all lesions by combining the diagnostic accuracies of each group after applying different cutoff values of T/N ratio, sensitivity (78.4%) and accuracy (79.2%) were somewhat improved compared to diagnostic values using non-corrected cutoff value of T/N ratio (70.6 and 74.0%, respectively). In particular, the specificity (80.8%) remained constant regardless of MTV correction (Table 3). A representative case, which had benefited from applying MTV-corrected cutoff values of T/N ratio on ^11^C-MET PET/CT for differential diagnosis, is shown in Fig. 1.

## Discussion

Early detection of recurrence after GKR was important for deciding the treatment strategy in patients with metastatic brain tumor. However, it is difficult to differentiate between radiation-induced necrotic inflammation and active tumor recurrence after treatment because necrotic lesions show similar enhancement on gadolinium-enhanced MRI [4, 11, 12]. ^11^C-MET PET/CT has played an important role in the diagnosis of tumor recurrence [1, 13], but it has limited application in evaluation of small lesions because of PVE, which results in underestimation of apparent ^11^C-MET activity [5, 7, 14]. These limitations of ^11^C-MET PET/CT could be critical in patients who underwent GKR, because it has often been used to treat small metastatic brain tumors (mostly less than 4 cm in maximal diameter) to avoid side effects such as radiation-induced inflammatory changes [4, 15].

The accuracy of ^11^C-MET PET/CT in distinguishing between recurrence and radiation necrosis has been variously reported as showing 60–100% sensitivity and 75–100% specificity in brain tumors [16]. Additionally, optimal cutoff value of T/N ratio, which was defined as maximal ^11^C-MET uptake in the lesion divided by that of the contralateral normal gray matter, has also been reported with variable range (1.4–1.9) in previous studies [2, 3, 6]. Our study showed that mean value of T/N ratio tended to decrease in both recurrence and radiation necrosis as tumor size decreased (Table 1). This suggested that different cutoff values of T/N ratio should be applied according to tumor size. However, the measured diameter on ^11^C-MET PET/CT might have poor inter-observer agreement. Tumor diameter on MRI does not represent the exact tumor burden because it includes both tumor recurrence and radiation necrosis. For that reason, it was very important to find a reproducible parameter to measure tumor size.

In several studies, MTV on ^11^C-MET PET/CT has been reported to represent the real tumor burden, which has been correlated with progression-free or overall survival in patients with brain tumor [13, 17]. Moreover, MTV has potential as a size criterion measured with reproducible manner, which applies a threshold of 1.3 times the mean ^11^C-MET uptake of contralateral normal gray matter. In our study, MTV was used as a size criterion for tumor grouping, derived from the diameter (1 and 2 cm) of the lesions that could be affected by PVE [5]. As a result, cutoff values of T/N ratio for diagnosis could be optimized in each group classified by MTV. In particular, in the group with MTV ≤ 0.5 cm^3^, there was no recurrent lesion with T/N ratio more than the non-corrected cutoff value (1.61). Conversely, in the group with MTV > 4.0 cm^3^, there was no radiation necrotic lesion with T/N ratio more than the MTV-corrected cutoff value (1.85) and no recurrent lesion with T/N ratio less than 1.61. This explains why McNemar’s test could not report the differences in diagnostic accuracies between the MTV-corrected and non-corrected cutoff values in each group.

Some necrotic lesions have also been reported to have a high ^11^C-MET uptake probably due to blood-brain barrier (BBB) disruption, inflammation, or reactive gliosis [18, 19]. Our study showed that T/N ratio of radiation necrosis also increased as MTV increased. Moreover, mean T/N ratio of radiation necrosis (1.65) was more than the non-corrected cutoff value (1.61) in the group with MTV > 4.0 cm^3^ (Table 2). Large necrotic tissues could have more false-positive uptakes of ^11^C-MET through variable factors mentioned above than small necrotic tissues, which could reduce the specificity of ^11^C-MET PET/CT. This could be another reason why application of different cutoff values of the T/N ratio according to tumor size is desirable for differential diagnosis between recurrence and radiation necrosis in metastatic brain tumor.

The T/N ratio could be also affected by factors other than tumor size. Pathophysiology of the necrotic lesion after therapy could be one of the possible reasons for the difference in cutoff values from previous studies, which showed that reactive gliosis was observed in necrotic tissues of gliomas but not of metastatic brain tumor [20, 21]. Terakawa et al. [2] showed that optimal cutoff values were different according to histologic types (1.41 for metastatic brain tumor vs. 1.58 for gliomas). Moreover, they also reported that ^11^C-MET uptake of normal gray matter in patients who underwent conventional radiotherapy tended to be lower than that in patients who underwent stereotactic radiosurgery. We additionally tried to evaluate whether T/N ratio was different according to histology of the primary lesion. When we compared the T/N ratios of recurrent lesions in metastatic lesions from the lungs, T/N ratio showed a variable range according to lung histology (adenocarcinoma: 2.10 ± 0.98; squamous cell carcinoma: 2.78 ± 1.18; small cell carcinoma: 1.74 ± 0.29), although the difference was not significant. Further studies are necessary to elucidate the relationship among T/N ratio, tumor histology, and tumor size.

There is no consensus regarding which parameter between SUVmax and mean standardized uptake value (SUVmean) of the normal gray matter on ^11^C-MET PET/CT is more useful to determine T/N ratio for differentiating between recurrence and radiation necrosis after radiation therapy in patients with brain tumors. Our study showed that the area under the ROC curve was 0.756 for T/N ratio used with SUVmean of the normal gray matter, which was lower than that used with SUVmax (0.790). In addition, the areas under the ROC curve of T/N ratio used with SUVmax were higher in each group classified by MTV. We adopted the following definition of T/N ratio: SUVmax of the lesion divided by that of normal gray matter. This may explain why optimal cutoff value of T/N ratio could be 1.23 in small lesions (MTV ≤ 0.5), although MTV was measured by applying a threshold 1.3 times the SUVmean of the normal gray matter from previous studies.

Compared to previous studies, our results showed relatively low negative predictive value (NPV). There could be several explanations. NPV was especially low in subgroups with small (MTV ≤ 0.5 cm^3^)- and large (MTV > 4.0 cm^3^)-sized lesions. Mean values of T/N ratio in both recurrence and radiation necrosis tended to be lower as tumor size decreased (Table 1). Low NPV could result from high prevalence of false-negative lesions although we had optimized cutoff value of T/N ratio in small sized tumors. Conversely, optimal cutoff value of T/N ratio in large-sized lesions was higher than that in all lesions from ROC curve analysis (1.85 vs. 1.61). High cutoff value of T/N ratio could also increase the false-negative results in large-sized lesions. Apart from diagnostic accuracy, several patients might be overdiagnosed to have recurrent lesions because 42 among 51 recurrent lesions were diagnosed with radiological tumor control after repeated GKR according to definition.

There were several limitations in this study. First, the retrospective nature of the study introduces the possibility of selection bias. Repeated GKR was done in patients with suspected recurrence, who seemed to lead a bias at the time of conducting ^11^C-MET PET/CT. The proportion between recurrence and radiation necrosis could affect optimal cutoff value of T/N ratio especially in small- or large-sized lesions. Second, the final diagnosis of radiation necrosis or tumor recurrence was performed mainly with radiological follow-up. The treatment effect of chemotherapy to control the primary tumor was not excluded. Third, a phantom experiment was not performed to evaluate PVE to affect T/N ratio in this study. Instead, we tried to set acceptable size criteria to measure MTV from previous study [5].

## Conclusions

Our study showed that optimal cutoff values of the T/N ratio should be different to maintain diagnostic accuracy regardless of tumor size. MTV has potential to be used as a reproducible parameter to reflect actual tumor size. Finally, MTV-corrected cutoff values of the T/N ratio can improve diagnostic performance of ^11^C-MET PET/CT without decreasing specificity especially in small lesions. We expect our results to contribute to reproducible and standardized interpretation of ^11^C-MET PET/CT.

## Abbreviations

^11^C-MET: ^11^C-methionine
CT: Computed tomography
GKR: Gamma knife radiosurgery
MRI: Magnetic resonance imaging
MTV: Metabolic tumor volume
PET: Positron emission tomography
PVE: Partial volume effect
ROC: Receiver operating characteristic
SUVmax: Maximum standardized uptake value
SUVmean: Mean standardized uptake value
T/N ratio: Tumor to normal tissue ratio
VOI: Volumetric region of interest
